# Repeated floating elbow injury after high-energy trauma

**DOI:** 10.1007/s11751-011-0102-7

**Published:** 2011-01-14

**Authors:** Olimpio Galasso, Massimo Mariconda, Giorgio Gasparini

**Affiliations:** 1 Department of Orthopaedic and Trauma Surgery, School of Medicine, Magna Græcia University, Campus S. Venuta—V.le Europa, 88100 Germaneto, Catanzaro, Italy; 2 Department of Orthopaedic and Trauma Surgery, School of Medicine, Federico II University, Naples, Italy

**Keywords:** Floating elbow injury, Recurrence of fractures, Open reduction and internal fixation, Difficult hardware removal

## Abstract

The floating elbow is an uncommon injury occurring both in children and in adults. Two reports of rare variants of floating elbow injury have been published, but to the best of our knowledge, no recurrence of this injury has been described. We present a complex pattern of floating injury, occurring in the same limb 3 years after a floating elbow lesion, which included supracondylar fracture of the humerus and associated ipsilateral midshaft fracture of forearm bones. Satisfactory outcomes were finally obtained. This clinical case illustrates the importance of carefully assessing floating elbow injuries when they occur to optimize the surgical strategies and the adequate timing of the treatment. A comprehensive literature review of the floating elbow injuries is here reported.

**Electronic supplementary material:**

The online version of this article (doi:10.1007/s11751-011-0102-7) contains supplementary material, which is available to authorized users.

## Introduction

The floating elbow, defined as a simultaneous ipsilateral fracture of the humerus and forearm, is an uncommon injury occurring both in children [1, 2] and in adults [3–6]. Two major categories of floating joint injuries have been described in the literature [4]: type-1 consisting of skeletal disruption above and below an articulation without direct injury to the intermediate joint and type-2 with combined skeletal and direct articular injury. A type-3 lesion, including associated neurovascular damage of overlying soft tissue elements, with or without simultaneous articular involvement, was later described [7]. Two reports of rare variants of floating elbow injury have been published [8, 9], but to the best of our knowledge no recurrence of this injury has been described. We present a complex pattern of injury, occurring in the same limb 3 years after the healing of a floating elbow lesion, which included supracondylar fracture of the humerus and associated ipsilateral midshaft fracture of forearm bones (i.e., iterative floating elbow injury). A comprehensive literature review of the floating elbow injuries and the critical management of this unique case are reported in the manuscript.

## Case report

**Fig. 1:**
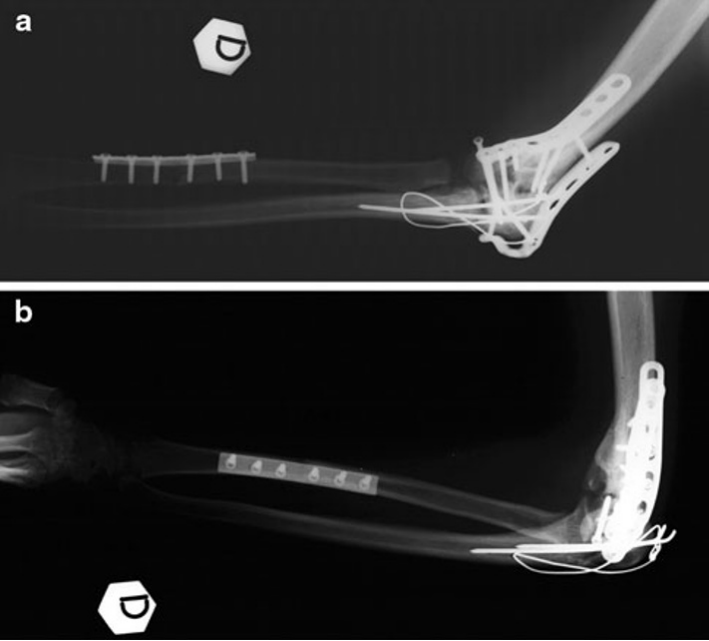
Plain radiographs (**a**, **b**) showing the healing of fractures 1 year after the first floating elbow injury

A 28-year-old man was admitted to our hospital because of a motorcycle trauma involving his right upper limb. Three years earlier, the patient had reported a type-1 floating elbow injury to the same limb from a motorcycle accident. The former lesion was treated at a different hospital by ORIF of both humerus and forearm fractures. In details, the comminuted intercondylar fracture was treated by two reconstructive plates on the medial and lateral columns of the distal humerus, along with interfragmentary screw fixation. The radial shaft fracture was treated by a six-hole plate with screws, whereas a tension band wiring was performed to stabilize the ulnar fracture. Radial head resection was also carried out (Fig. 1). Following this procedure, the patient obtained a painless elbow although the range of motion (i.e., 40° of extension, 90° of flexion, and 40° of forearm’s pronation/supination) and limb strength were reduced.

The patient refused further operations to improve articular range of motion, thus living with this function for 3 years prior to the second high-energy trauma to the elbow.

**Fig. 2:**
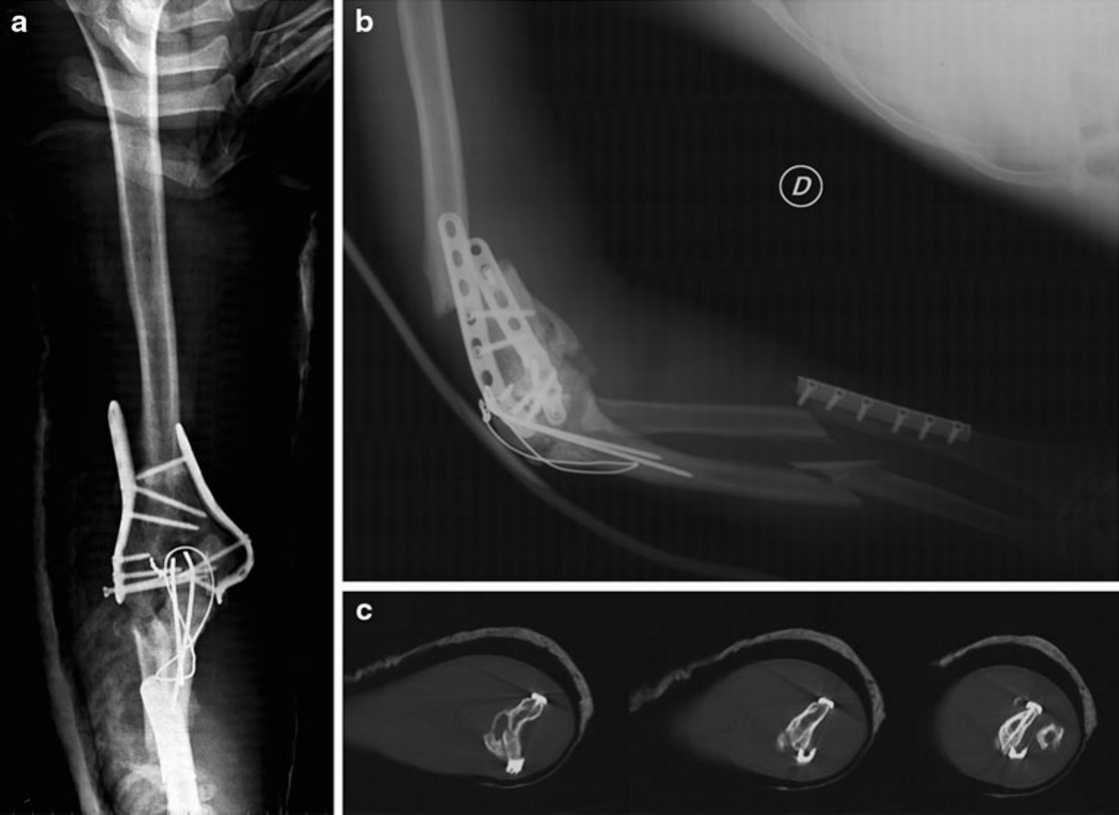
AP (**a**) and lateral (**b**) radiographs at hospital admittance show supracondylar fracture of the humerus and midshaft fractures of radius and ulna (second floating elbow injury). The CT scan (**c**) of distal humerus demonstrates the intercondylar integrity with an intra-articular bridge resulting from the previous treatment

When the patient was admitted at our emergency room, the physical examination showed bruising, severe soft tissue swelling, and gross deformity of the right elbow and forearm. He complained of tingling in his forearm and inability to carry out active movement of his right hand. The neurological examination showed severe tactile hypoesthesia and paresis of the muscles in the radial and ulnar nerve territories. No anomalies in the arterial pulses were detected. The patient also reported facial soft tissue injury and non-nasal midfacial fractures. The radiographic examination showed a supracondylar fracture of the humerus and midshaft fractures of the radius and ulna. Thus, according to Simpson and Jupiter [7], a type-3 floating elbow injury was diagnosed. In detail, a fracture of the humerus close to the most proximal screw occurred, the ulna had a fracture distally to the former fracture with an intermediate third fragment, while the radius sustained a fracture at the site of the most proximal screw (Fig. 2a, b). A CT scan (Aquilion 64-slice CT, Toshiba Corporation, Japan) showed integrity of the intercondylar bone, with the implants of the former operation stable (Fig. 2c).

**Fig. 3:**
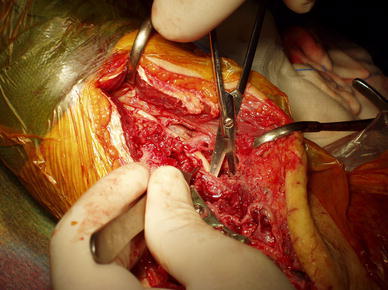
The radial nerve is proximally dissected so that its course on the posterior side of the humerus is clearly identified and the Y plate can be safety positioned

**Fig. 4:**
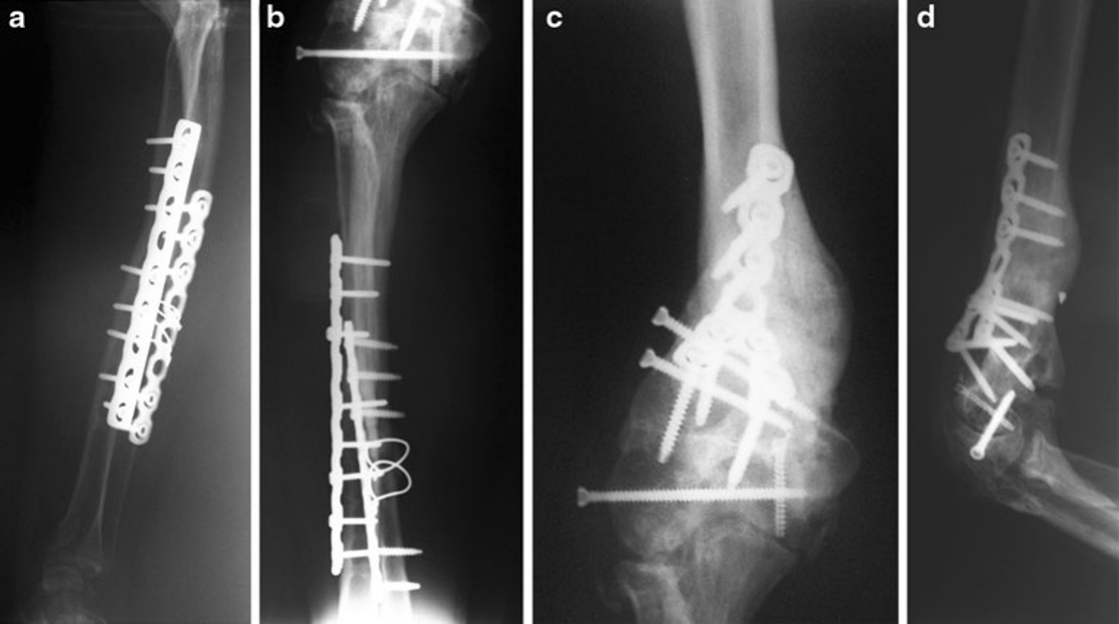
Anteroposterior and lateral radiographs showing healing of forearm (**a**, **b**) and humeral (**c**, **d**) fractures 1 year postoperatively

**Fig. 5:**
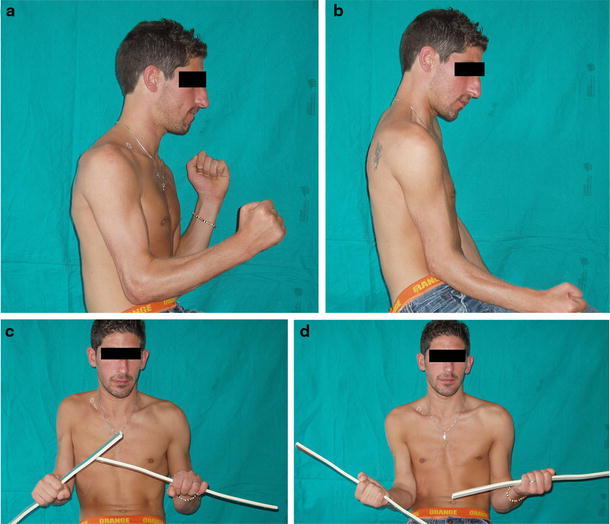
Photographs (**a**, **b**, **c**, **d**) showing active motion of the right elbow in comparison with the uninjured contralateral limb 18 months after the second injury

The treatment of fractures was delayed by 3 days because of tissue swelling. A dissection of an inverted V-shaped flap of the triceps aponeurosis that was then reflected distally was the approach used for the reconstruction of the fractured distal humerus. The ulnar nerve was therefore mobilized and allowed to remain in its normal anatomical position, but it was carefully protected until the end of surgical procedure. The radial nerve was also dissected and fully mobilized (Fig. 3) to avoid its improper stretching caused by muscle spreading at the time of plate positioning. The radial and the ulnar nerves were intact, and no signs of compression by the fracture fragments were noted. A dorsolateral approach to the radius and a lateral approach to the ulna were chosen for the surgical exposure of the forearm. All previous metal implants except two interfragmentary screws and two screws fixing the lateral plate to the humerus were removed. The removal of these screws was unfeasible, and therefore, the lateral plate was cut with metal-cutting saw. In the operative theater, the fractures that had occurred during the first floating elbow injury appeared completely healed. An ORIF of the humeral and forearm fractures using one Y-plate and two straight plates was respectively performed. Cable wires were used to stabilize the third ulnar fragment. The postoperative period was uneventful, and the patient was encouraged to start exercising the shoulder and the hand the day after surgery. The elbow was immobilized in a plaster cast for 3 weeks to limit active movements. Rehabilitation of the elbow was then begun. Clinical and roentgenographic follow-up controls were performed at 1, 3, 6, and 18 months. A functional assessment was made using the Liverpool Elbow Score (LES) that had been previously validated and tested for its internal consistency [10]. The LES includes a nine-item patient-answered questionnaire and six surgeon-oriented items, with a total score ranging from 0 (worst) to 10 (best). The patient’s fractures partially consolidated within 3 months and radiological evaluation 1 year after surgery showed the union of all fractures (Fig. 4). At 18-month follow-up, the LES score was 5.72 and the patient had reduced but painless elbow range of motion compared to the opposite side (Fig. 5). The elbow showed 90° of flexion and 45° of extension, the pronation and supination of the forearm were 55° and 45°, respectively, and the palmar grip strength, when measured with a static strength tester (CSD 300 Chatillon—Ametek Inc., Florida), was 50% with respect to the uninjured side. Electroneuromyography revealed progressive improvement in the radial and ulnar nerves since the sixth-month follow-up examination, but at the final evaluation, residual reduction of electrophysiological parameters compared to normal values was recorded in the ulnar nerve territory. The patient was satisfied with the functional result and refused to undergo further surgery to improve the elbow motion. For the present case report, the patient was informed that data concerning the case would be submitted for publication and gave his consent.

## Discussion

Floating elbow is an uncommon injury in both children [1, 2] and adults [3–6], and to the best of our knowledge, no recurrence of this injury in the same limb has been described in the literature available. We reported a case of recurrent floating elbow occurring in a previously injured limb with an 18-month follow-up. Our patient first sustained a type-2 floating injury, and 3 years later, a type-3 lesion occurred in the same elbow.

The patient’s history could explain the mechanism of reinjury. Indeed, he sustained a fall on the outstretched hand with the wrist dorsiflexed, the forearm pronated, and the elbow partially extended. The radius fractured cephalad to the proximal end of the plate used to fix the former fracture, and the ulna fractured in its midshaft. Thus, a peri-plate fracture of the forearm due to stress shielding following the high-energy injury occurred. The resultant moment of force caused an extension-type supracondylar fracture of the distal humerus. This latter fracture occurred at the site of insertion of the most proximal screw of the plate used 3 years earlier. Indeed, the characteristics of the current fractures were likely influenced by the change in the stress distribution pattern caused by the pre-existing hardware. Moreover, the initial treatment of the humeral fracture with little fixation proximal to the fracture site despite quite a length of plate available for fixation is questionable. Clinical indications for implant removal are not well established [11], and in the case here reported, there is uncertainty whether the implant removal would be appropriate after the healing of fractures of first elbow injury [12]. The removal of the hardware after union of fractures would have caused a different pattern of bony lesions even though it is nearly impossible to prefigure the nature of these fractures. At the time of admission, the CT examination was crucial to plan the appropriate surgical technique and the high-resolution images showed the integrity of the intercondylar bone, thus allowing the safety removal of previous implants. As for the type of fixation, plate fixation is recommended in floating elbow injuries [7] and is the gold standard method, even for the most complex forearm fractures [13]. We used a short Y-plate on the humerus to obtain adequate control of the fracture fragments avoiding extensive soft tissues dissection. This procedure led to fracture healing, despite it was a rather instable reconstruction. Indeed, a longer plate should have been selected mainly for the fixation of the diaphyseal fragment to reduce the period of postoperative immobilization. Indeed, in primary cases, a shorter immobilization period (i.e., 1 week after surgery) has been recommended [5]. However, the cautionary choice of a 3-week postoperative plaster cast can be warranted because of the complexity of this rare iterative injury.

A limited contact dynamic compression plate was used to fix the radius, whereas a reconstruction plate was used as a bridged plate to treat the multifragmentary ulnar fracture.

Although our patient was satisfied with the final functional result of surgery, the range of motion of his elbow and mainly the extension was limited. A poor functional outcome is common after floating elbow injury [5, 14], and most of the functional impairment detected in our patient after the treatment for the recurrent trauma was caused by the former injury. Indeed, limitation of elbow range of motion and weakness of the overall limb were already present at the time of the second injury. Actually, it would have been impossible to obtain significant increase in the elbow functionality with the repeated ORIF because of the long-lasting restriction in ROM. Several complications can occur after floating elbow injury, e.g., infection, myositis ossificans, non-union, and malunion of the humerus or forearm bones, as well as vascular or nerve injury, leading to poor functional results. One previous study on twenty-one patients with floating elbow indicated that only 28% of them had good results, with residual neurologic dysfunction in more than 50% [14]. Indeed, the association of a neurovascular injury adversely affects the functional outcome after trauma to the upper limb [13], and nerve injury represents a negative outcome predictor in the floating elbow [5, 6]. In our patient, the ulnar nerve deficit partially recovered at the 18 months. Nevertheless, he was satisfied with his care and refused further surgery.

The present case draws attention to the floating elbow injuries. This is the first description of a case of recurrence of such lesion. The report suggests that even if a high-energy trauma to the elbow of our patient had occurred after a first floating elbow injury, a satisfactory result can be achieved with an open reduction and internal fixation of fractures combined with an early course of physical therapy. Preoperative CT imaging and nerve studies can aid for an accurate diagnosis and are advised to plan the final treatment of a recurrent floating elbow injury. The removal of the hardware after bone healing could be advised dealing with young patients sustaining similar injuries with multiple fractures.

## Electronic supplementary material

Below is the link to the electronic supplementary material.

Supplementary material 1 (JPEG 3022 kb)

Supplementary material 2 (JPEG 2516 kb)
