# Supplementary figures and images for: Repeated floating elbow injury after high-energy trauma
Source: Strategies Trauma Limb Reconstr. 2011 Jan 14;6(1):33–7. doi: 10.1007/s11751-011-0102-7 (PMC3058185; doi:10.1007/s11751-011-0102-7)

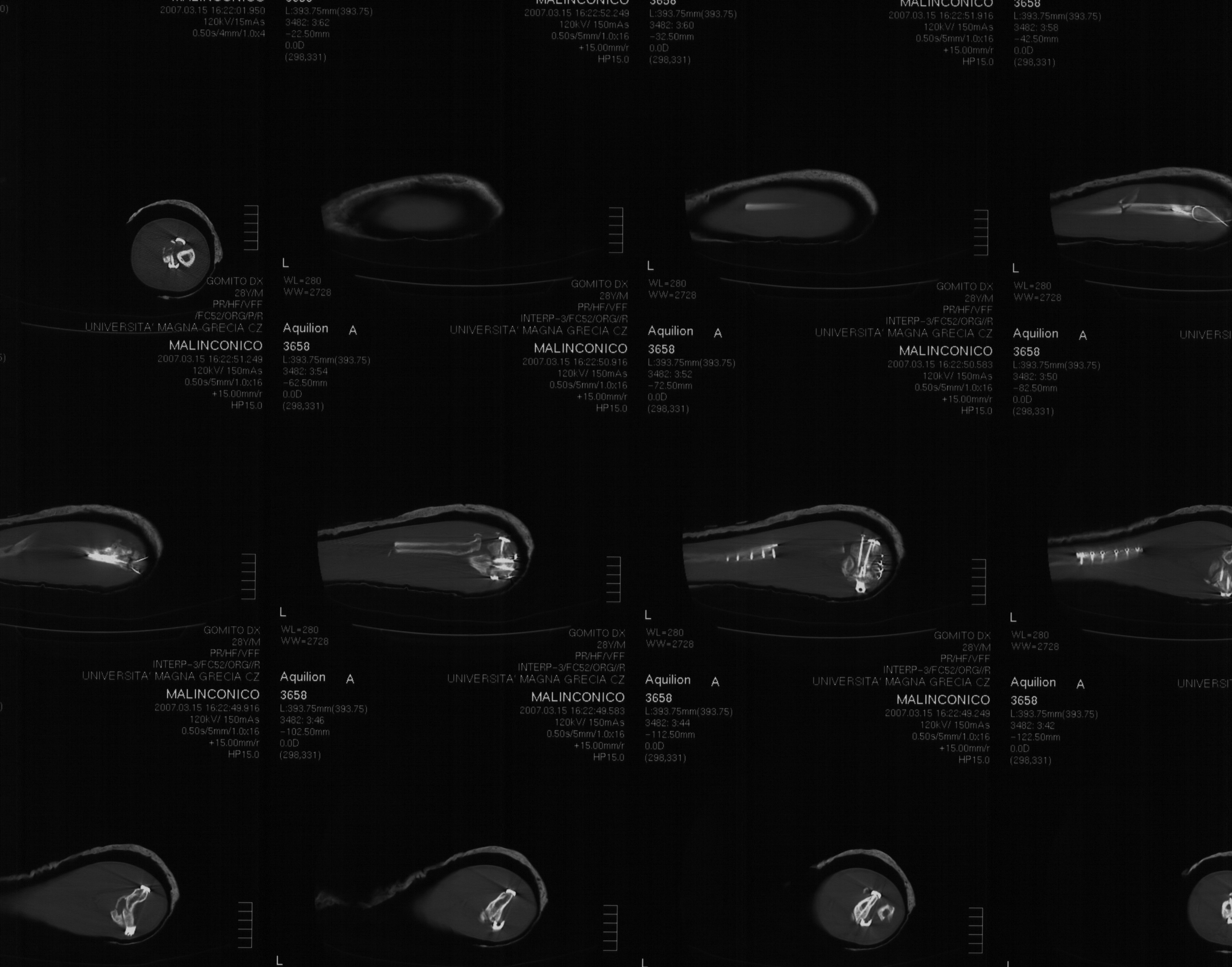

Supplement: Supplementary file 1 — Supplementary material 1 (JPEG 3022 kb) [file 11751_2011_102_MOESM1_ESM.jpg]

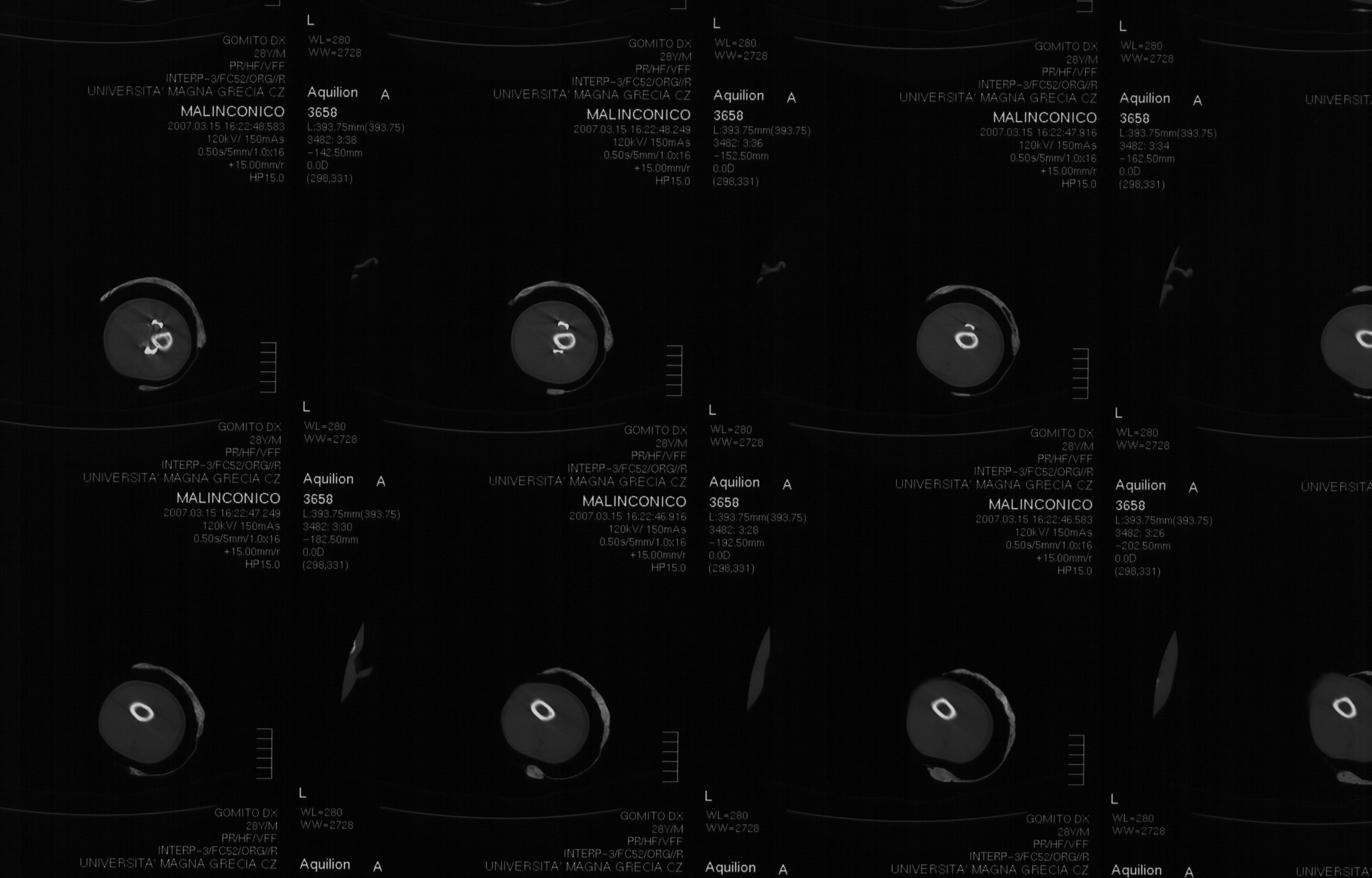

Supplement: Supplementary file 2 — Supplementary material 2 (JPEG 2516 kb) [file 11751_2011_102_MOESM2_ESM.jpg]
